# Combined TGF-β3 and FGF-2 Stimulation Enhances Chondrogenic Potential of Ovine Bone Marrow-Derived MSCs

**DOI:** 10.3390/cells14131013

**Published:** 2025-07-02

**Authors:** Sandra Stamnitz, Agnieszka Krawczenko, Aleksandra Klimczak

**Affiliations:** Laboratory of Biology of Stem and Neoplastic Cells, Hirszfeld Institute of Immunology and Experimental Therapy, Polish Academy of Sciences, 53-114 Wroclaw, Poland; agnieszka.krawczenko@hirszfeld.pl (A.K.); aleksandra.klimczak@hirszfeld.pl (A.K.)

**Keywords:** ovine mesenchymal stem cells, chondrogenic differentiation, signaling molecules, cartilage regeneration, large animal model

## Abstract

Mesenchymal stem cells (MSCs) represent a promising cell source for cartilage tissue engineering due to their chondrogenic potential. However, current differentiation protocols result in limited efficiency. This study assessed the combined effects of transforming growth factor-beta 3 (TGF-β3) and fibroblast growth factor-2 (FGF-2) on the morphology, proliferation, chondrogenic differentiation, chondrogenic gene expression, and cytokine profile of ovine bone marrow-derived MSCs (BM-MSCs). BM-MSCs were cultured under four conditions: control (αMEM) or αMEM supplemented with FGF-2, TGF-β3, or TGF-β3 + FGF-2. Morphological and proliferation analyses, Alcian blue staining in 2D and 3D, and real-time PCR for early (*Chad*, *Comp*, and *Sox 5*) and late (*Agg*, *Col IX*, *Sox 9*, and *Fmod*) chondrogenic markers were performed. Cytokine secretion profiles were analyzed using multiplex assay. TGF-β3 induced morphological changes indicative of early chondrogenesis, while FGF-2 enhanced proliferation. The combination of both cytokines led to a synergistic increase in cell proliferation, early and late chondrogenic gene expression, and glycosaminoglycans (GAG) deposition. Cytokine analysis revealed that TGF-β3 enhanced the immunomodulatory and angiogenic profile of BM-MSCs, whereas co-treatment with FGF-2 yielded a balanced and potentially regenerative secretome. Dual stimulation with TGF-β3 and FGF-2 significantly improves the chondrogenic differentiation of ovine BM-MSCs by enhancing both molecular and functional markers of cartilage formation.

## 1. Introduction

Articular cartilage has a low capacity for self-repair because it is avascular, aneural, and alymphatic in nature. Damages to this specialized tissue, whether by trauma or by degenerative diseases, for example osteoarthritis, often lead to progressive dysfunctions of joint and pains [1]. Conventional clinical interventions, including microfracture, autologous chondrocyte implantation, and osteochondral grafting, provide short- to mid-term symptomatic reliefs but rarely bring regeneration of hyaline-like cartilage with correct biomechanical and biochemical properties [2]. Therefore, there is a significant need for regenerative approaches, which will promote stable and functional cartilage repair. Mesenchymal stem cells (MSCs) are becoming a promising cell source for cartilage tissue engineering because of their ability for self-renewal, their immunomodulatory properties, and their capacity for differentiation into chondrocytes under proper stimulation [3]. MSCs can be isolated from various tissues, like bone marrow, adipose tissue, and synovium, but bone marrow-derived MSCs (BM-MSCs) are one of the most frequently studied [4]. Moreover, it has been demonstrated that MSCs from different tissue sources exhibit varying chondrogenic potential, proliferation rates, and responsiveness to growth factors, which may significantly affect their suitability for cartilage regeneration strategies. Bone marrow-derived MSCs show a strong baseline potential for chondrogenic differentiation; however, selection of the culture conditions remains crucial for achieving consistent outcomes in tissue regeneration [5]. Despite their inherent chondrogenic potential, MSCs cannot easily differentiate into stable, mature chondrocytes in vitro and in vivo without external stimulation. Also, their differentiation is often accompanied by unwanted hypertrophic changes, which finally lead to endochondral ossification instead of keeping stable cartilage tissue [6]. The success of MSC-based regenerative strategies also strongly depends on the interaction between cells, bioactive factors, and scaffold materials, which collectively influence differentiation efficiency and tissue integration [7]. To remove these limitations, many studies have focused on optimizing the microenvironment and biochemical signals that control MSCs behavior [3]. One of the most important factors are members of the transforming growth factor-beta (TGF-β) family, especially TGF-β3, which is known for starting early chondrogenic commitment and facilitating upregulation of key cartilage matrix components like aggrecan through Smad-dependent signaling [8]. However, stimulation with only TGF-β is not enough to guarantee long-term keeping of chondrogenic phenotype or stopping hypertrophic gene expressions like collagen type X [9]. Fibroblast growth factor 2 (FGF-2) is the next signaling molecule that garners great interest because of its ability to expand MSCs and keep them multipotent and sensitive to chondrogenic stimuli. It was shown that FGF-2 can increase the expression of Sox9, the main regulator of chondrogenesis, and can create a stronger effect of TGF-β3 for promoting cartilage matrix production [10]. While both factors alone influence MSCs, some studies suggest that when combined or used one after the other, they give synergistic effects and result in more effective and stable chondrogenic differentiation [11].

As for the validation of regenerative strategies before clinical use, one important thing in translational research is using MSCs that come from large animals. Although human MSCs are well characterized for chondrogenic differentiation [12,13], we chose ovine BM-MSCs because our ultimate goal is to translate the in vitro findings into in vivo applications in a large animal model. In comparison to rodents, large animals like sheep, goats, and pigs have cartilage dimensions, joint loads, and healing reactions more like humans. Using MSCs from large animals gives better prediction value for in vitro and in vivo experiments and makes a smaller gap between laboratory and therapy for humans [14]. In vivo studies in an animal model are essential before this therapy can be applied in humans, as they enable the development of an optimized and safe treatment protocol [15]. Ovine BM-MSCs are a special good platform for checking cartilage repair methods before clinical translation because of the anatomical and biomechanical similarities of their joint to the human knee [16]. Even if there is much evidence supporting the single roles of TGF-β3 and FGF-2, there is still a limited number of complete studies that examine together the morphology, proliferation, molecule profiles, and secretions of MSCs after this combined stimulation in the context of cartilage regeneration. Therefore, the aim of this study was to investigate the effects of TGF-β3 and FGF-2, both alone and together, on the morphology of ovine BM-MSC, their proliferation, expressions of chondrogenic genes, deposition of glycosaminoglycans with Alcian blue staining in 2D and 3D cultures, and cytokines secretion profile. This integrative method gives a better understanding of how these growth factors influence MSC chondrogenesis and help build better regenerative strategies for cartilage repair. Using ovine MSCs here gives useful insights that are important for real clinical cartilage repairs for humans.

## 2. Materials and Methods

### 2.1. Cell Culture

Mesenchymal stem cells were isolated from sheep bone marrow as previously described [17]. Cells were cultured in Minimum Essential Medium α-transformation (αMEM) (IIET PAS, Wroclaw, Poland) supplemented with 10% fetal bovine serum (FBS) (Biowest, Riverside, Montana, MT, USA, cat. no. S1810-500), 2 mM L-glutamine (Biowest, Riverside, Montana, MT, USA, cat. no. X0550-100), and 1% penicillin/streptomycin (Merck, Saint Louis, MO, USA, cat. no. P0781) at 37 °C and 5% CO2. Bone marrow-derived mesenchymal stem cells (BM-MSCs) were passaged with Accutase Cell Detachment Solution (Corning, Manassas, VA, USA, cat. no. 25-058-CI) and used for experiments at passage 2 or 3. Four different cell culture media were used to analyze chondrogenic differentiation: (1) αMEM (control), (2) αMEM supplemented with 20 ng/mL FGF-2 (Merck, Saint Louis, MO, USA, cat. no. F0291), (3) αMEM supplemented with 10 ng/mL TGF-β3 (ImmunoTools GmbH, Friesoythe, Germany, cat. no. 11343153), and (4) αMEM supplemented with both 20 ng/mL FGF-2 and 10 ng/mL TGF-β3. The control and supplemented media were changed three times a week. The experiments on chondrogenic-induced BM-MSCs were performed on days 7, 14, and 21 of incubation.

### 2.2. Proliferative Activity

To assess the proliferation rate of ovine BM-MSCs under the influence of FGF-2 and TGF-β3, the MTT assay was conducted. Cells were seeded at a density of 2 × 10^3^ cells per well in 96-well plates and cultured in αMEM or αMEM supplemented with either FGF-2, TGF-β3, or a combination of both. The MTT assay was performed after 4, 24, 48, 72, and 96 h of cell incubation. At each time point, 10 µL of a 4 mg/mL MTT solution (Merck, Saint Louis, MO, USA, cat. no. M2128) was added to each well and incubated for 4 h at 37 °C. After incubation, the medium was removed, and 100 µL of DMSO (POCh, Gliwice, Poland, cat. no. 363550117) was added to dissolve the formazan crystals. The absorbance was measured at 570 nm using Wallac Victor2 microplate reader (Perkin Elmer LAS, Waltham, MA, USA). The doubling time (DT) of BM-MSCs was calculated based on the growth curves obtained from the MTT assay results, using the following formula:

(1) $$DT=T\cdot \frac{ln2}{ln\frac{N_{T}}{N_{0}}}$$

where T is the incubation time (hours), N_T_ is the number of cells after the incubation time, and N_0_ is the number of cells initially harvested. Three independent MTT assays were conducted to obtain the cell doubling time.

### 2.3. Alcian Blue Quantification in 2D and 3D Culture

The chondrogenic differentiation of ovine BM-MSCs treated or untreated with FGF-2 and TGF-β3 was evaluated with the use of Alcian blue staining in two different cell culture formats: 2D monolayer culture in well plates and 3D micropellet culture in V-shaped well plates. For 2D monolayer culture, 100 µL of 5 × 10^3^ cells were added to each well of a 96-well plate. After an overnight attachment, the medium was replaced with the chondrogenic differentiation medium (PromoCell, Heidelberg, Germany, cat. no. C-28016) either supplemented or not with FGF-2 or TGF-β3 or both. For 3D micropellet culture, 100 µL of a 5 × 10^5^ cells/mL cell suspension was added to each well of the V-shaped 96-well plate (Sarstedt, Nümbrecht, Germany). Plates were spun in a centrifuge at 500× *g* for 5 min to pellet the cells, and after overnight incubation, the media was replaced with 200 mL of each treatment condition. To avoid cell pellet disruption, 70% of culture media was replaced three times a week throughout the 21 days of the culture period. After 7, 14 and 21 days, each culture condition in 2D and 3D was washed with PBS and fixed in a 3.7% formaldehyde (Merck, Saint Louis, MO, USA, cat. no. 104003) for 20 min at RT. Each well was rinsed with PBS again and incubated with 100 µL of Alcian blue for 40 min (Merck, Saint Louis, MO, USA, cat. no. A3157). After multiple PBS washes, 100 µL of PBS was added before imaging with a Primovert inverted microscope (Zeiss, Jena, Germany). To quantify the staining, PBS was removed, and bound Alcian blue was extracted at RT using 100 µL of 6 M guanidine hydrochloride on a plate shaker for 2 h. The quantification was achieved using a Wallac Victor2 microplate reader at 650 nm absorption.

### 2.4. Real-Time PCR

To examine the effect of TGF-β3 and FGF-2 treatment of sheep BM-MSCs on the expression level of early chondrogenic differentiation genes: *chondroadherin* (*Chad*), *cartilage oligomeric matrix protein* (*Comp*)*,* and *SRY-Box Transcription Factor 5* (*Sox 5*) and late chondrogenesis genes, namely *aggrecan* (*Agg*), *collagen type IX* (*Col IX*), *SRY-Box Transcription Factor 9* (*Sox 9*), and *fibromodulin* (*Fmod*), real-time polymerase chain reaction (real-time PCR) was conducted. BM-MSC were cultured for 21 days in (1) control αMEM, (2) αMEM with FGF-2, (3) αMEM with TGF-β3, and (4) αMEM with FGF-2 and TGF-β3. At three time points, namely 7, 14, and 21 days, total ribonucleic acid (RNA) was isolated using the NucleoSpin^®^ RNA Kit (Macherey-Nagel, Düren, Germany, cat. no. 740955.50) according to the manufacturer’s instructions. Then, 1 µg of total RNA from each sample was reverse-transcribed to prepare cDNA using the RevertAid First Strand cDNA Synthesis Kit (Thermo Fisher, Vilnius, Lithuania, cat. no. K1622). Real-time PCR was performed using Power SYBR Green PCR Master Mix (Life Technologies, Warrington, UK, cat. no. 4367659) and analyzed using the ViiA 7 Real-Time PCR system (Applied Biosystems, Foster City, CA, USA). Reactions were performed three times with two biological replicates with the following program settings: initial denaturation at 95 °C for 10 min, followed by 40 cycles of denaturation at 95 °C for 15 s, annealing at Tm (°C) of the primers listed in Table 1 for one minute, and extension at 72 °C for 40 s. Data were analyzed by calculating the fold differences in gene expressions of the treated cells compared to untreated cells after they were normalized to their own GAPDH housekeeping gene value.

### 2.5. Cytokine Array

The C-Series Ovine (Sheep) Cytokine Array C1 Kit (Ray-Bio^®^, Norcross, GA, USA) was used to assess the effect of TGF-β3 and FGF-2 on the cytokine profile of ovine BM-MSCs. This kit is based on a semi-quantitative protein array method and allows the detection of multiple proteins in a single assay. Cells were cultured in αMEM supplemented with 2 mM L-glutamine and 1% penicillin/streptomycin without FBS (1) and with (2) FGF-2, (3) TGF-β3, (4) FGF-2, with TGF-β3 for 7, 14, and 21 days. First, cell cultures were established in T75 culture flasks at a density of 1.9 × 10^4^ cells/cm^2^ in a complete αMEM medium with FBS and incubated for 24 h. The medium was then changed to αMEM without FBS with or without TGF-β3 and/or FGF-2. The medium was changed every three days. After the appropriate time points, supernatants were collected in 15 mL tubes and centrifuged at 1200 rpm for 10 min at room temperature and then placed in new tubes and centrifuged again at 1600 rpm for 10 min to remove contaminants. The secretory profile of sheep BM-MSC was examined using the semiquantitative C-Series Ovine (Sheep) Cytokine Array C1 Kit (Ray-Bio^®^, Norcross, GA, USA, cat. no. AAO-CYT-1-8) according to the manufacturer’s protocol. Briefly, membranes were placed in incubation troughs and incubated at room temperature for 30 min with 2 mL of blocking buffer. The buffer was then removed, and 1 mL of supernatants was added and incubated overnight at 4 °C. The next step was to wash the membranes and incubate them with 1 mL of biotinylated antibody cocktail for 2 h at room temperature. A second wash was performed to remove unbound antibody, after which the membranes were incubated with 1 mL of horseradish peroxidase-streptavidin conjugate at room temperature for 2 h. Finally, the membranes were washed, and chemiluminescent detection was performed using X-ray film. Differences in relative protein expression were measured using ImageJ (version 1.53k, US National Institutes of Health, Bethesda, MD, USA) with the Protein Array Analyzer plugin. The data were then analyzed in Microsoft Excel-based analytical software for the Ovine Cytokine Array C1 kit (Ray-Bio^®^, Norcross, GA, USA). The results are presented on heat maps.

### 2.6. Statistical Analysis

All graphs and statistical analysis were performed using GraphPad Prism vision 10.04 (GraphPad Software, Inc., San Diego, CA, USA). One-way ANOVA analysis was performed using Dunnett’s test for multiple comparison procedures. Error bars on graphs show the standard error of the mean. *p*-values < 0.05 were considered as statistically significant.

## 3. Results

### 3.1. Effect of TGF-β3 and FGF-2 on Sheep BM-MSCs Morphology and Proliferation

**Figure 1:**
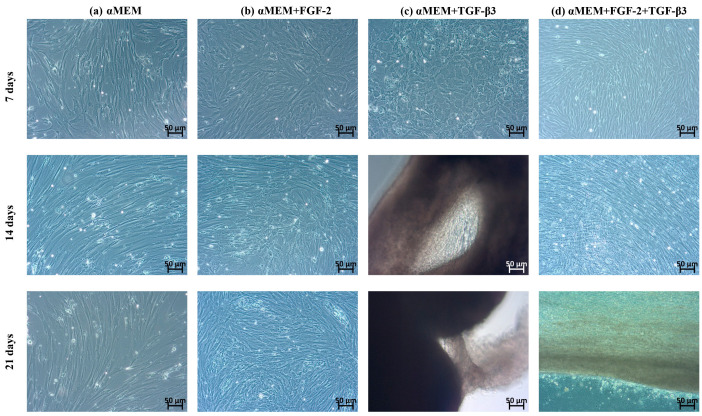
Representative images demonstrate morphological changes of (**a**) untreated sheep BM-MSCs and those (**b**) treated with FGF-2, (**c**) TGF-β3, and (**d**) FGF-2 combined with TGF-β3 for 7, 14, and 21 days. BM-MSCs treated with TGF-β3 exhibit a distinct morphology compared to other conditions, resembling a cobblestone-like appearance.

In αMEM culture medium without cytokine addition, BM-MSCs showed spindle-shaped morphology, typical of MSCs (Figure 1a). Similarly, in αMEM medium with the addition of FGF-2—the cells were spindle-shaped but smaller, and after 21 days of incubation, their number was significantly higher than in the control medium (Figure 1b). The greatest morphological differences were observed in cell culture treated with TGF-β3, with cells adopting an irregular shape resembling cobblestone morphology, which is characteristic of primary chondrocytes. After 14 and 21 days of culture in medium supplemented with TGF-β3, the cells formed dense aggregates (Figure 1c). When BM-MSCs were cultured in medium supplemented with both FGF-2 and TGF-β3 (Figure 1d), no significant changes in cell shape were observed, but an accelerated proliferation rate was noted after just 7 days of culture compared to cells untreated or treated with the cytokines separately.

**Figure 2:**
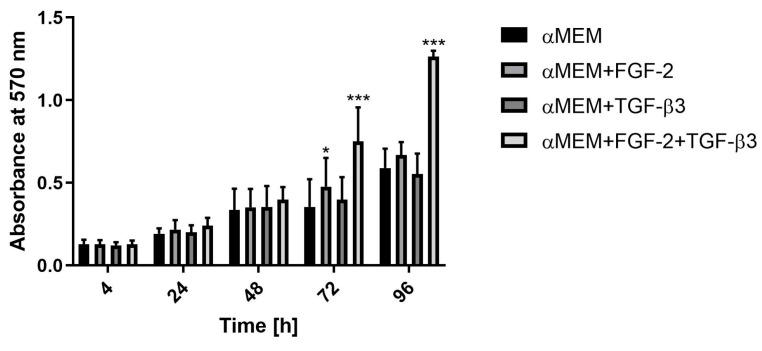
Effect of cytokines FGF-2 and TGF-β3 on proliferation of sheep BM-MSCs. * *p* < 0.05; *** *p* < 0.0001. Three independent experiments in triplicates were performed. FGF-2 in combination with TGF-β3 most significantly enhances the proliferation of ovine BM-MSCs.

The proliferation rate of sheep BM-MSCs untreated or treated with FGF-2 and TGF-β3 cytokines was evaluated using the MTT assay. Cells cultured in αMEM medium and in αMEM supplemented with FGF-2 and/or TGF-β3 cytokines had similar proliferation rates up to 48 h. However, significant differences were observed after 72 and 96 h of incubation, where BM-MSC cells cultured in medium supplemented with both cytokines FGF-2 and TGF-β3 showed the highest proliferative activity (Figure 2). Cells treated with TGF-β3 alone had similar proliferation rates to untreated cells throughout the observation period. In contrast, BM-MSCs treated with FGF-2 had a higher proliferation rate than cells cultured in medium without the addition of cytokines. BM-MSCs doubled in cell number most rapidly when treated simultaneously with FGF-2 and TGF-β3 under a doubling culture time of ~ 20 h and most slowly in control medium ~ 27 h (Table 2).

### 3.2. Comparing 2D and 3D Chondrogenic Differentiation

**Figure 3:**
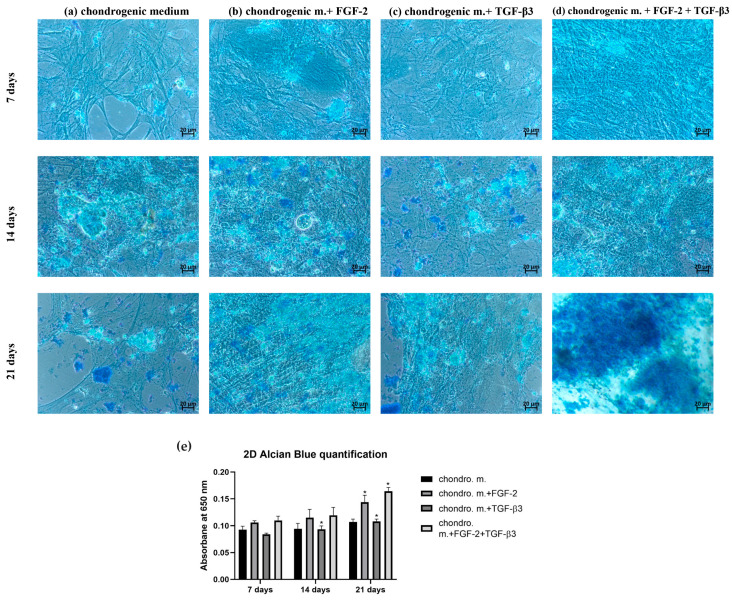
Alcian blue staining of ovine BM-MSCs in 2D culture in (**a**) chondrogenic differentiation medium (**b**) with FGF-2, (**c**) TGF-β3, (**d**) FGF-2, and TGF-β3. (**e**) Effect of TGF-β3 and FGF-2 cytokines on the intensity of Alcian blue staining in 2D culture for 21 days of culture. Three independent experiments in triplicates were performed. * *p* < 0.05. Ovine BM-MSCs treated with FGF-2 and TGF-β3 exhibit the highest chondrogenic differentiation potential.

**Figure 4:**
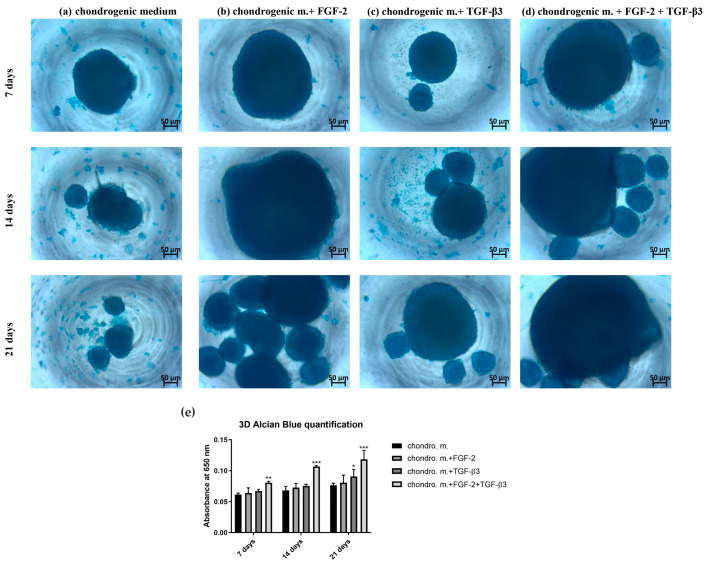
Alcian blue staining of ovine (BM-MSCs) in 3D culture in (**a**) chondrogenic differentiation medium (**b**) supplemented with FGF-2 (**c**) TGF-β3, (**d**) FGF-2, and TGF-β3. (**e**) Intensity of Alcian blue staining of sheep BM-MSCs in 3D culture for 21 days. Three experiments in triplicates were performed. * *p* ˂ 0.05, ** *p* ˂ 0.005, and *** *p* ˂ 0.0001. Treatment of sheep BM-MSCs with both FGF-2 and TGF-β3 enhances 3D chondrogenic differentiation.

Chondrogenic differentiation confirmed by Alcian blue staining was performed under 2D (Figure 3) and 3D (Figure 4) conditions to more accurately represent in vivo conditions. In 2D culture, sheep BM-MSCs were observed to differentiate into chondrogenic cell lines after only 7 days of incubation in differentiation medium. However, the intensity of staining was higher for cells cultured in differentiation medium supplemented with FGF-2 or FGF-2 and TGF-β3 (Figure 3b,d) compared to BM-MSCs untreated with cytokines or treated with TGF-β3 alone (Figure 3a,c). To verify the chondrogenic potential depending on the differentiation medium (with or without cytokines) and in particular whether the addition of both cytokines FGF-2 and TGF-β3 results in greater chondrogenic differentiation efficiency than the addition of single cytokines, Alcian blue extraction was performed, and absorbance was measured. After 21 days of culture, sheep BM-MSCs treated with FGF-2 and TGF-β3 showed the highest chondrogenic differentiation capacity (Figure 3e). Also, addition of FGF-2 cytokine alone significantly increased the chondrogenic potential of sheep BM-MSCs. In contrast, the addition of TGF-β3 cytokine alone did not increase the intensity of staining compared to the use of differentiation medium alone without additives.

For 3D chondrogenic differentiation, different-sized cell pellets were observed depending on the addition or absence of cytokines in the differentiation medium (Figure S1 in Supplementary Materials presents the surface area of the pellets depending on the culture conditions and incubation time). The largest cell aggregates of sheep BM-MSCs were observed when chondrogenic medium was used with the addition of FGF-2 alone or FGF-2 and TGF-β3 (Figure 4b,d). Interestingly, after 21 days, a clear difference in the size of cell aggregates was seen between BM-MSCs cultured in medium with TGF-β3 alone and BM-MSCs in differentiation medium without the addition of cytokines (Figure 4a,c). Also, in the quantitative analysis, the higher intensity of Alcian blue staining of sheep BM-MSCs cultured in differentiation medium with the addition of TGF-β3 compared to BM-MSCs not treated with any cytokines was confirmed. Nevertheless, as in the case of 2D culture, the highest capacity for chondrogenic differentiation of BM-MSCs cultured in medium with the addition of both cytokines FGF-2 and TGF-β3 was also observed under 3D conditions (Figure 4e).

### 3.3. Chondrogenic Gene Markers Expression

To evaluate the effects of TGF-β3 and FGF-2 on the early and late stages of chondrogenesis in sheep BM-MSCs, we examined the expression of genes of early markers of chondrogenic differentiation, namely *chondroadherin* (*Chad*), *cartilage oligomeric matrix protein* (*Comp*), and *Sox 5*, and genes of the late stage of chondrogenesis, namely *aggrecan* (*Agg*), *collagen IX* (*Col IX*), *Sox 9*, and *fibromodulin* (*Fmod*), after 7, 14, and 21 days of culture in medium with or without TGF-β3 and FGF-2.

**Figure 5:**
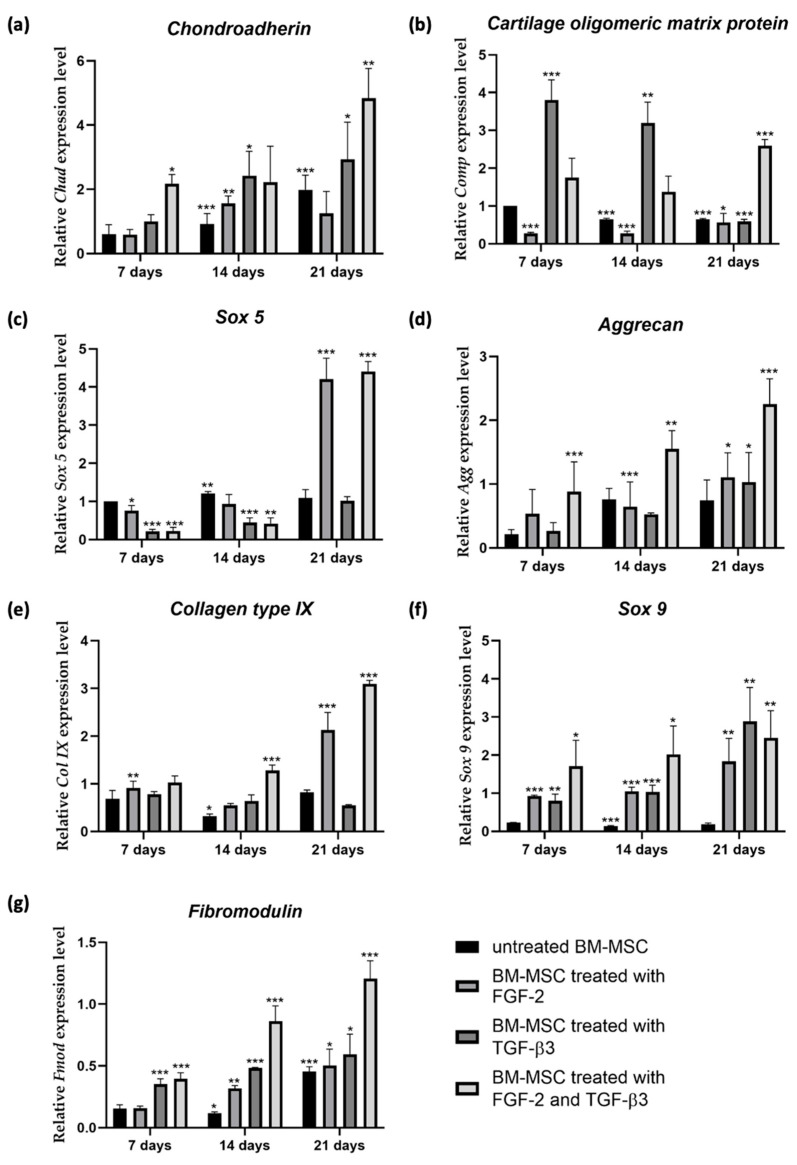
Real-time PCR analysis for markers of chondrogenic differentiation of sheep BM-MSCs treated with TGF-β3 and/or FGF-2 for 7, 14, and 21 days. *Chad*, *Comp*, and *Sox 5* (**a**–**c**) characterize the early stage of chondrogenesis; gene expression of *Agg*, *Col IX*, *Sox 9*, and *Fmod* (**d**–**g**) characterize the late stage of chondrogenic differentiation. * *p* ˂ 0.05, ** *p* ˂ 0.005, and *** *p* ˂ 0.0001. Three experiments with two biological replicates were conducted. Treatment of ovine BM-MSCs with the cytokines FGF-2 and TGF-β3 increase the expression of both early and late chondrogenic marker genes.

The lowest levels of chondroadherin expression throughout the entire incubation period of the cells (7, 14, and 21 days) were shown by untreated sheep BM-MSCs, while BM-MSCs treated with FGF-2 showed a moderate increase in *Chad* expression but not as pronounced as with TGF-β3. BM-MSCs treated with TGF-β3 had significantly higher levels of expression, especially on the later days of the experiment. Interestingly, *Chad* expression was highest in cells treated with a combination of FGF-2 and TGF-β3, suggesting a synergistic effect of these two cytokines in promoting gene expression (Figure 5a). Sheep BM-MSCs treated with FGF-2 showed the lowest levels of relative *Comp* expression throughout the experiment. In contrast, the highest level of *Comp* expression was observed for BM-MSCs treated with TGF-β3 at the early stage of chondrogenesis after 7 days. After 14 days, *Comp* expression under these conditions decreased slightly, but by day 21, a significant reduction in the relative expression level of this gene was observed. After 21 days of BM-MSC culture, the highest level of *Comp* expression was observed for cells treated with both cytokines (Figure 5b). The highest levels of *Sox 5* expression were observed after 21 days of BM-MSC culture treated with FGF-2 alone or FGF-2 in combination with TGF-β3. For the other time points (7 and 14 days), the relative expression level of this gene was very low for all culture conditions (Figure 5c).

Relative gene expression levels of the late-stage chondrogenesis marker *Agg* increased over time for all culture conditions tested but were highest for sheep BM-MSCs treated with both FGF-2 and TGF-β3 cytokines, while BM-MSCs treated separately with FGF-2 or TGF-β3 at day 21 had similar levels of relative *Agg* expression (Figure 5d). The highest levels of *collagen IX* expression were observed after 21 days of culture for BM-MSCs treated with both cytokines FGF-2 and TGF-β3 and slightly lower levels for BM-MSCs treated with FGF-2 alone. In contrast, when cells were treated with TGF-β3 alone, *Col IX* expression levels were very low at each observation point (Figure 5e). Another marker gene of late stages of chondrogenesis, *Sox 9*, for all culture conditions was at similar levels after 7 and 14 days of incubation. The increase in *Sox 9* expression was not apparent until day 21 of culture and was highest for BM-MSCs treated with TGF-β3 alone (Figure 5f). The relative level of *fibromodulin* expression increased over time for all culture conditions and was highest for BM-MSCs treated with FGF-2 and TGF-β3 (Figure 5g).

### 3.4. Influence of TGF-β3 and FGF-2 on Ovine BM-MSCs Cytokine Profile

**Figure 6:**
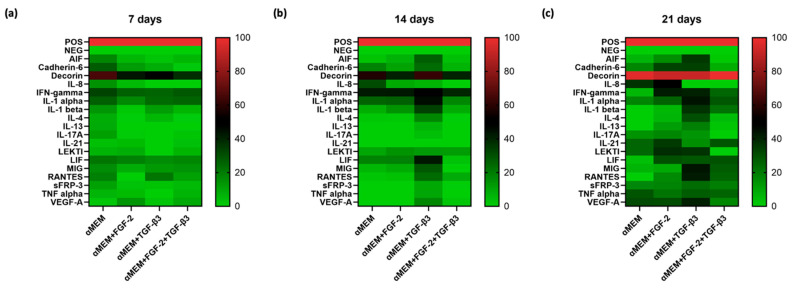
Cytokine profile of sheep BM-MSCs after stimulation with FGF-2, TGF-β3, or FGF-2 and TGF-β3 or no cytokine addition at three time points: (**a**) 7 days, (**b**) 14 days, or (**c**) 21 days. Supplementation of ovine BM-MSCs with FGF-2 and TGF-β3 affects their cytokine profile.

Using the semi-quantitative C-Series Ovine (Sheep) Cytokine Array C1 Kit, the expression levels of 18 ovine cytokines were analyzed in supernatants from BM-MSCs cultured in the media: (a) αMEM, (b) αMEM supplemented with FGF-2, (c) αMEM supplemented with TGF-β3, and (d) αMEM supplemented with FGF-2 and TGF-β3. This analysis was performed to determine the effect of the tested cytokines on the secretion profile of sheep BM-MSCs at 7, 14, and 21 days (Figure 6a–c). It was shown that under all culture conditions, decorin, a cytokine that influences fibrillogenesis, had the highest relative expression levels, which increased over time and, after 21 days of culture, accounted for approximately 100% of the internal positive control. The expression levels of the cytokines tested increased over time for all culture media. However, the highest number of cytokines at significantly higher expression levels after 21 days of incubation was observed in the supernatant of cells cultured in αMEM medium supplemented with TGF-β3. Thirteen out of the eighteen cytokines tested showed expression levels above 25%, among which immunomodulatory and proangiogenic cytokines were the most abundant, such as decorin (~91%), interleukin 1 alpha (IL-1α) (~44%), interferon gamma-induced monokine–MIG (~43%), vascular endothelial growth factor A (VEGF-A) (~41%), RANTES (~41%), interferon gamma (IFNγ) (~40%), interleukin 1 beta (IL-1β) (~37%), LEKTI (~36%), transplant inflammatory factor (AIF) (~35%), cadherin 6 (~33%), interleukin-4 (IL4) (~30%), leukemia inhibitory factor (LIF) (~28%), and tumor necrosis factor (TNFα) (~27%). Analyzing the 21st day of culture, it was noted that treatment of sheep BM-MSCs with FGF-2 alone or FGF-2 in combination with TGF-β3 also influenced higher levels of cytokine expression in culture medium compared to control conditions (culture in αMEM). In the culture medium of cells treated with FGF-2, nine cytokines showed expression levels above 25%: decorin (~92%), interleukin 8 (IL-8) (~50%), IFNγ (~42%), LEKTI (~38%), interleukin 21 (IL-21) (~35%), cadherin 6 (~33%), VEGF-A (~31%), LIF (~30%), and IL-1α (~26%). In contrast, when cells were treated with FGF-2 and TGF-β3, expression levels above 25% were recorded for seven cytokines: decorin (~100%), LIF (~30%), IL-21 (~29%), IL-1α (~29%), MIG (~28%), RANTES (~26%), and TNFα (~25%). For sheep BM-MSCs cultured in αMEM, only five cytokines showed expression levels above 25%: decorin (~97%), IL-8 (~37%), VEGF-A (~32%), TNFα (~29%), and LEKTI (~25%).

## 4. Discussion

Mesenchymal stem cells (MSCs) are famous for their capacity to differentiate into chondrocytes, making them promising candidates for cartilage tissue engineering. However, the efficiency of MSC chondrogenesis still remains suboptimal, often resulting in the development of cartilage with limited functions. A significant problem is the tendency of MSCs to go into hypertrophic differentiation, which can result in endochondral ossification and worsen the quality of regenerated cartilage [18]. To make better chondrogenic potential of MSCs, this study was focused on using specific growth factors supporting proper chondrogenesis. Transforming growth factor-beta 3 (TGF-β3) has been indicated as an important regulator, promoting the expression of chondrogenic markers like aggrecan through Smad2/3 pathways [19]. At the same time, fibroblast growth factor 2 (FGF-2) activates the MAPK/ERK and PI3K/Akt pathways via FGFR1/2, which not only support proliferation of MSCs but also enhance early expression of the chondrogenic transcription factor Sox 9 [20]. When used in combination, FGF-2 and TGF-β3 exhibit a synergistic effect on MSC chondrogenesis. FGF-2 primes MSCs for chondrogenesis via Sox 9 early appearance [21], and this priming amplifies the effect of TGF-β3, which upon binding its receptors activates Smad2/3 signaling, further enhancing Sox 9, aggrecan, and cartilage oligomeric matrix protein expression [19]. Moreover, the proliferative boost from FGF-2 helps to increase the number of progenitor cells, which can be used later for differentiation, and it is often used for improving cartilage tissue engineering protocols [10,11]. Despite this progress, the combined effect of TGF-β3 and FGF-2 for MSC chondrogenesis is still poorly explored. This study aimed to investigate if applying TGF-β3 and FGF-2 together can improve the chondrogenic differentiation of ovine bone marrow-derived MSCs in vitro.

In the first stage of research, the morphological and proliferative responses of ovine BM-MSCs to FGF-2, TGF-β3, and their combination were observed. In the control medium (αMEM), BM-MSCs showed typical fibroblast-like, spindle-shaped morphology consistent with the phenotype of undifferentiated MSCs. After FGF-2 stimulation, cells kept a spindle shape but appeared smaller and more densely packed, showing a better proliferation rate. This observation corresponds with previous studies that FGF-2 supports MSCs proliferation [22]. In contrast, TGF-β3 made big morphological changes. Cells became smaller with irregular shapes and formed dense groups resembling cobblestone morphology, typical for early chondrogenic commitment. These changes fit the known function of TGF-β3 for supporting chondrogenic differentiation, including cell condensation and changes in cytoskeleton organization [23]. Interestingly, when both FGF-2 and TGF-β3 were added together, BM-MSCs did not show a cobblestone morphology as with TGF-β3 alone but still had a spindle shape similar to the FGF-2 group. Even without clear morphological signs of chondrogenesis, proliferation was the biggest in the combined application of FGF-2 and TGF-β3, with a very fast doubling time (~20 h). This suggests that FGF-2 can regulate morphological changes resulting from the action of TGF-β3 and together can enhance proliferation. These observations are in line with those of Solchega et al. and Correa et al., who showed that FGF-2 makes MSCs better prepared to respond to TGF-β3, demonstrating a synergistic interaction between these growth factors [10,21]. Overall, our data confirm that combining FGF-2 and TGF-β3 can help control MSC proliferation and morphology more efficiently for cartilage engineering.

The microenvironment plays an important role in guiding the chondrogenesis of MSCs. Traditional 2D cultures are very often used because of their easy manipulation, but they are not good copies of the 3D structure and mechanical forces of real cartilage. Thus, 2D culture can limit the study of the real effectivity of chondrogenic differentiation. Three-dimensional culture systems offer an environment more like real tissue [24,25,26]. Alcian blue staining, which shows how much glycosaminoglycans (GAGs) are deposited, is a key marker of effective chondrogenesis. In our study, differences in GAG deposition were observed between 2D and 3D cultures, showing how TGF-β3 and FGF-2 affect chondrogenic differentiation of MSCs. In the 2D model, TGF-β3 alone caused moderate GAG deposition, as confirmed by Alcian blue staining, proving its ability to start chondrogenic differentiation, which this is in line with earlier results [27]. FGF-2 alone did not strongly improve GAG deposition, matching its primary role for cell proliferation, not differentiation. However, a combination of TGF-β3 and FGF-2 resulted in significantly stronger Alcian blue staining than single-agent treatments. This suggests synergy: FGF-2 primes MSCs to be more sensitive to TGF-β3. Indeed, it was found that MSCs expansion in FGF-2-supplemented medium elevates the baseline levels of the chondrogenic master regulator Sox 9, which together with the sustained proliferative state result in early and enhanced chondrogenic differentiation once TGF-β3 is applied [21]. Switching to 3D cultures, it gives even greater chondrogenic effects. The most effective GAG deposition was observed in the TGF-β3 + FGF-2 group as evidenced by Alcian blue staining. The 3D culture better supports cell–cell and cell–matrix interactions necessary for mature chondrogenesis. While TGF-β3 starts chondrogenesis, FGF-2 improves MSC proliferative condition, and the 3D environment helps stabilize cartilage-like tissue formation [21,28,29]. Our study indicates the high importance of combining biochemical factors and culture dimensionality for successful cartilage tissue engineering.

It is well known that chondrogenic differentiation of MSCs is controlled by transcription factors and external signals regulating specific cartilage genes [30]. We analyzed the expression of important chondrogenic genes after stimulation with TGF-β3, FGF-2, and both. Different combinations of cytokine treatments showed different gene expression patterns, proving complex regulation of chondrogenesis. Early markers of chondrogenesis, like *chondroadherin* (*Chad*) and *cartilage oligomeric matrix protein* (*Comp*), were strongly upregulated by the application of TGF-β3 alone; however, in combination with FGF-2, their expression was much stronger. These results prove the main role of TGF-β3 in starting chondrogenesis. FGF-2 alone only slightly affects early chondrogenic genes expression, showing its mitogenic and not chondrogenic role. However, combined treatment has been showing to strongly upregulate the expression of *Chad* and *Comp*. According to current knowledge, there are no published studies specifically investigating the synergistic effect of FGF-2 and TGF-β3 on the expression of *Comp* and *Chad* genes. However, researchers have reported higher expression of these genes when MSCs were treated with TGF-β3 [31,32]. Expression of *Sox 5*, important for chondrogenic commitment, was very high in the FGF-2 group at day 21. In the combined application of FGF-2 and TGF-β3, the *Sox 5* expression level was also very high. FGF-2 is known for upregulating Sox genes and preparing MSCs for differentiation [21]. Late markers, like *aggrecan* (*Agg*) and *fibromodulin* (*Fmod*), showed the strongest expression under combined treatment of FGF-2 and TGF-β3. Scientists have shown that adding TGF-β3 results in a significant increase in *aggrecan* expression compared to untreated MSCs [33]. *Collagen type IX* (*Col IX*) was at the highest expression level under combined treatment of FGF-2 and TGF-β3. *Sox 9*, the main chondrogenic transcription factor, was mostly induced by TGF-β3, but the combination of FGF-2 and TGF- β3 also kept a high *Sox 9* level after 21 days. This shows that FGF-2 does not block chondrogenesis but rather supports it by proliferation and priming [21]. Altogether, our gene expression results show that TGF-β3 is the main driver of chondrogenesis, while FGF-2 enhances MSCs proliferation ability, thereby amplifying the overall chondrogenic outcome.

The secretion profile of MSCs is also important, showing their regenerative and immunomodulatory potential. After 21 days, we measured a panel of cytokines secreted by ovine BM-MSCs under different treatment conditions. TGF-β3-treated cells presented the highest cytokine diversity, including high IL-1α, IL-1β, IFN-γ, VEGF-A, RANTES, and LIF. FGF-2 treatment alone increased IL-8, IL-21, and VEGF-A, supporting the healing process. A combination of FGF-2 and TGF-β3 showed a balanced cytokine profile. Although the cytokines number was a bit lower than when TGF-β3 alone was applied, important factors like LIF, MIG, RANTES, and TNF-α were still at a high level. Decorin, an important matrix proteoglycan, was expressed at the highest level in all conditions, confirming its role in cartilage matrix stability [34]. Overall, TGF-β3 is a strong stimulator of bioactive factors secretion by MSC, but the addition of FGF-2 better regulates this process, providing a controlled environment for tissue regeneration. Considering morphology, proliferation, and chondrogenic gene expression, our cytokine profile data demonstrate the advantage of using both agents, FGF-2 and TGF-β3, to improve the cartilage engineering procedure.

Based on the experimental timeline applied in this study—7, 14, and 21 days of chondrogenic differentiation—our results demonstrate that the most pronounced chondrogenic effects were observed at day 21. While early responses such as morphological changes, modest increases in GAG deposition and gene expression of chondrogenic markers were evident by day 14, and after 21 days of culture, the combination of FGF-2 and TGF-β3 led to the strongest enhancement of chondrogenesis and a favorable cytokine secretion profile associated with cartilage regeneration. This time-dependent progression underscores the importance of sustained stimulation to achieve effective chondrogenic differentiation and supports the use of a 21-day culture period as optimal for evaluating the synergistic effects of FGF-2 and TGF-β3 in ovine BM-MSCs. These findings are consistent with other studies on MSC chondrogenesis, in which 21 days of culture is also commonly reported as the optimal time point for observing robust chondrogenic differentiation [13,35].

## 5. Conclusions

This study demonstrates that the combined stimulation of ovine BM-MSCs with TGF-β3 and FGF-2 produces a synergistic effect that enhances chondrogenic differentiation. While TGF-β3 alone induces early chondrogenic commitment and upregulates key matrix-related genes, and FGF-2 promotes MSC proliferation and priming, their combination significantly improves both molecular and functional markers of cartilage formation. Notably, this dual stimulation boosts glycosaminoglycan (GAG) deposition in both 2D and 3D culture conditions and enhances the expression of both early (*Chad*, *Comp*, and *Sox 5)* and late (*Agg*, *Col IX*, *Fmod*, and *Sox 9*) chondrogenic markers. Moreover, the combined treatment modulates the MSC secretory profile, supporting a balanced immunomodulatory and pro-regenerative environment that may favor stable cartilage repair. These findings not only confirm the complementary roles of TGF-β3 and FGF-2 in regulating MSC fate but also emphasize the importance of combining specific biochemical cues to overcome current limitations in MSC-based cartilage engineering. Altogether, this study provides a strong foundation for further translational research and suggests that TGF-β3 and FGF-2 co-treatment may represent an effective strategy to enhance the quality and stability of engineered cartilage for clinical applications.

## Figures and Tables

**Table 1:** Primers list.

Gene	Full Name	Sequences (5′-3′)	Tm (°C)	Fragment Size (bp)
*Chad*	*Chondroadherin*	F: CATCTGGAGAACAACCGCCT R: GGGCGAGAGGTCTTAGCTTC	60	146
*Comp*	*Cartilage oligomeric matrix protein*	F: GGCAGCAGGTCAAGGAGATT R: GTCTCGGTACAAGCCACTCC	60	154
*Sox 5*	*SRY-Box Transcription Factor 5*	F: AGAAGGCAGAAGAAGGTGGG R: AGTTCCCTGATCCCATTGCA	58	222
*Agg*	*Aggrecan*	F: GACTGTGAGGTACCCCATCC R: TCCGGGGATGTTGCATAGAA	60	165
*Col IX*	*Collagen type IX*	F: TGCCGACGGATTAACAGGT R: AAGCCAATTGTTCCACTGGG	58	243
*Sox 9*	*SRY-Box Transcription Factor 9*	F: TGAATCTCCTGGACCCCTTC R: CTTGTCCTCCTCGCTCTCCT	60	203
*Fmod*	*Fibromodulin*	F: TGACAATCGCAACCTCAAGTA R: TTGTTGTGGTCCAGGTACAG	58	222

**Table 2:** Population doubling time of ovine BM-MSCs depending on the growth medium used.

Culture Medium	Doubling Time [h]
αMEM	27.49 ± 0.56
αMEM+FGF-2	22.32 ± 0.42
αMEM+TGF-β3	25.32 ± 0.36
αMEM+FGF-2+TGF-β3	20.23 ± 0.37

## Data Availability

All data generated or analyzed during this study are included in this article.

## Supplementary Materials

The following supporting information can be downloaded at https://www.mdpi.com/article/10.3390/cells14131013/s1. Figure S1: Surface area of cell pellets formed during 3D chondrogenic differentiation of ovine BM-MSCs.
