# Supplementary material for: Combined TGF-β3 and FGF-2 Stimulation Enhances Chondrogenic Potential of Ovine Bone Marrow-Derived MSCs
Source: Cells. 2025 Jul 2;14(13):1013. doi: 10.3390/cells14131013 (PMC12249412; doi:10.3390/cells14131013)
Supplement: Supplementary file 1 [file cells-14-01013-s001.zip › cells-3689021-supplementary.pdf]

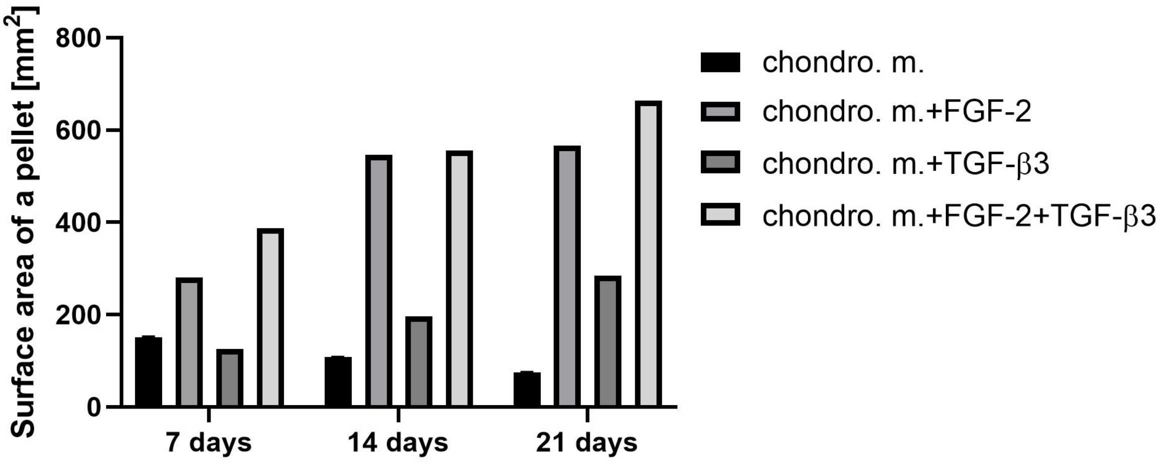

Figure S1. Surface area of cell pellets formed during 3D chondrogenic differentiation of ovine BM-MSCs.
